# Shortsightedness

**Published:** 1885-01

**Authors:** 


### Shortsightedness.

A writer in the London *Times* claims the cause of myopia to be the application of the eyes to near objects; in other words, the poring over books and handicrafts. When the eyes are directed to a near object, they are turned in or rendered convergent, so that the axes of vision meet upon it, and this position is maintained by a muscular effort which, if continued, alters the shape of the eye in the direction of elongation. Manifestly the alteration will be most easily affected during youth, when the tissues of the body, including those of the eye, are comparatively lax and distensible, and it will also be most easily effected among those young people whose tissues are exceptionally weak, by reason of inadequate food or of unhealthy descent or surroundings. Badly lighted schools are the great manufactories of myopia, the bad light compelling approximation of the books or other materials of study.

ABSORBANT TOW.—Signor Silvio Plevani, Director of the Hospital Frate Bene Fratelli, published in the *Gazzetta degli Ospitali*, of May 28, a paper on "Economical Preparation of some Antiseptic Dressings." He states that tow, which is a very cheap residual material, can be used for all surgical purposes instead of absorbent cotton, when prepared according to the following directions:

Boil the tow for some time in lye made from wood ashes, or in a ten per cent solution of corbonate of sodium, then wash it repeatedly in water. The tow, thus deprived of grease, is immersed in a ten per cent. solution of chloride of lime, and kept in it some hours, with occasional stirring, until it has become perfectly white. It is then washed thoroughly in pure water, until the liquid sqeezed from it is perfectly limpid. Drying and carding complete the process.—*The Lancet.*

